# Aspirin in the Modern Era of Cardiovascular Disease Prevention

**DOI:** 10.14797/mdcvj.293

**Published:** 2021-09-24

**Authors:** Ella Murphy, James M. G. Curneen, John W. McEvoy

**Affiliations:** 1 National University of Ireland, Galway, Ireland

**Keywords:** aspirin, antiplatelet, preventive cardiology, cardiovascular disease

## Abstract

Aspirin’s antithrombotic effects have a long-established place in the prevention of cardiovascular disease (CVD), and its traditional use as a core therapy for secondary prevention of CVD is well recognized. However, with the advent of newer antiplatelet agents and an increasing understanding of aspirin’s bleeding risks, its role across the full spectrum of modern CVD prevention has become less certain. As a consequence, recent trials have begun investigating aspirin-free strategies in secondary prevention. For example, a contemporary metanalysis of trials that assessed P2Y_12_ inhibitor monotherapy versus prolonged (≥ 12 months) dual antiplatelet therapy (which includes aspirin) after percutaneous coronary intervention reported a lower risk of major bleeding and no increase in stent thrombosis, all-cause mortality, myocardial infarction (MI), or stroke in the P2Y_12_ monotherapy group.

In contrast to secondary prevention, aspirin’s role in primary prevention has always been more controversial. While historical trials reported a reduction in MI and stroke, more contemporary trials have suggested diminishing benefit for aspirin in this setting, with no reduction in hard outcomes, and some primary prevention trials have even indicated a potential for harm. In this review, we discuss how changing population demographics, enhanced control of lipids and blood pressure, changes in the definition of outcomes like MI, evolution of aspirin formulations, and updated clinical practice guidelines have all impacted the use of aspirin for primary and secondary CVD prevention.

## Introduction

Studied in one of the first randomized clinical trials in medical history, aspirin is one of the oldest and most well-known medications in Western medicine.^1^ Due to its proven efficacy, aspirin has been called a “wonder drug.”^2^ Irreversibly inhibiting cyclooxygenase and thereby decreasing platelet aggregation, aspirin’s antithrombotic effects quickly established it as a staple in the prevention of cardiovascular disease (CVD).^3,4^ However, with advances in other treatment areas and changing population demographics, its role in cardiovascular disease prevention is evolving. In this focused review, we provide an update of aspirin’s role in the primary and secondary prevention of CVD, with some insights into where the field of CVD prevention might be heading next. The role of aspirin in the treatment and secondary prevention of stroke is not discussed. As such, our focus is on aspirin use among patients at risk for, or with a history of, either acute coronary syndrome (ACS) or chronic coronary syndrome (CCS).

## Secondary Prevention

Aspirin is a generally unquestioned core therapy in the secondary prevention of CVD because it inhibits platelet aggregation, thereby reducing the risk for recurrent arterial thrombosis.^3,4^ With a strong evidence base confirmed by the Antithrombotic Trialists’ Collaboration, international guidelines recommend lifelong aspirin as secondary prevention for the majority of adults at risk for recurrent CVD.^3,4,5,6,7^ Even so, the advent of newer and more potent antiplatelet drugs, such as the P2Y_12_ inhibitors, have expanded antithrombotic options for secondary prevention beyond aspirin.^8,9^

**Figure 1:**
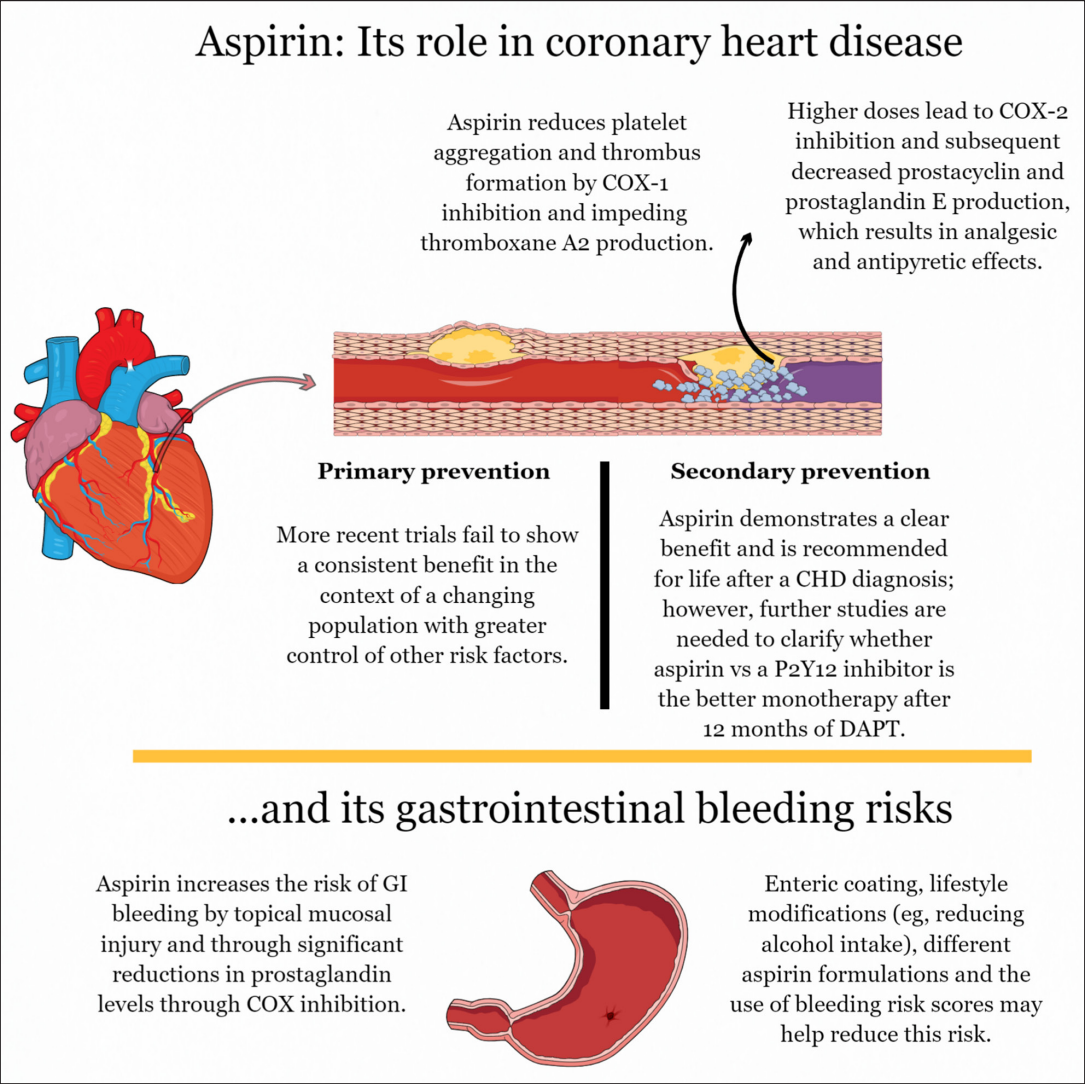
The role of aspirin in primary and secondary prevention. COX: cyclooxygenase isoenzyme; CHD: coronary heart disease; P2Y_12_: a chemoreceptor for adenosine diphosphate; DAPT: dual antiplatelet therapy; GI: gastrointestinal.

Aspirin’s association with an increased bleeding risk is also well established, particularly with respect to gastrointestinal bleeding events (***Figure 1***). While many of these events are not fatal, bleeding linked to antiplatelet use after percutaneous coronary intervention (PCI) has been associated with an increased risk of all-cause mortality.^10^ Therefore, recent trials have increasingly tested so-called “aspirin-free” strategies in selected secondary prevention patients. We note here that the term “aspirin-free strategy” is technically a misnomer when applied to persons undergoing PCI since no outcomes trial to date has tested the efficacy and safety of PCI without providing aspirin and another antiplatelet agent at the time of PCI and immediately afterwards. Rather, almost all of these “aspirin-free strategy” trials tested discontinuing aspirin 1 or more months after PCI, with only one trial studying PCI with aspirin loading provided at the time of the procedure but no aspirin provided afterwards.^11^ As such, we are not aware of any trial in which patients undergoing PCI received no periprocedural aspirin at all.

### Aspirin in Patients with Recent Percutaneous Coronary Intervention

As noted above, aspirin’s role in the immediate period (1–3 months) after recent PCI remains unquestioned to date. The traditional approach was to continue a regimen of oral dual antiplatelet therapy, or DAPT (such as aspirin plus another platelet inhibitor), for a period of 6 or 12 months after PCI in CCS and ACS, respectively. Following that, guidelines recommend lifelong aspirin therapy.^12^ However, the ubiquitous requirement for post-PCI aspirin beyond the 1- to 3-month mark has recently been challenged. Recent trials have started to examine both shorter durations of DAPT as well as monotherapy with P2Y_12_ inhibitors in place of aspirin, particularly in the setting of triple therapy.

**Figure 2:**
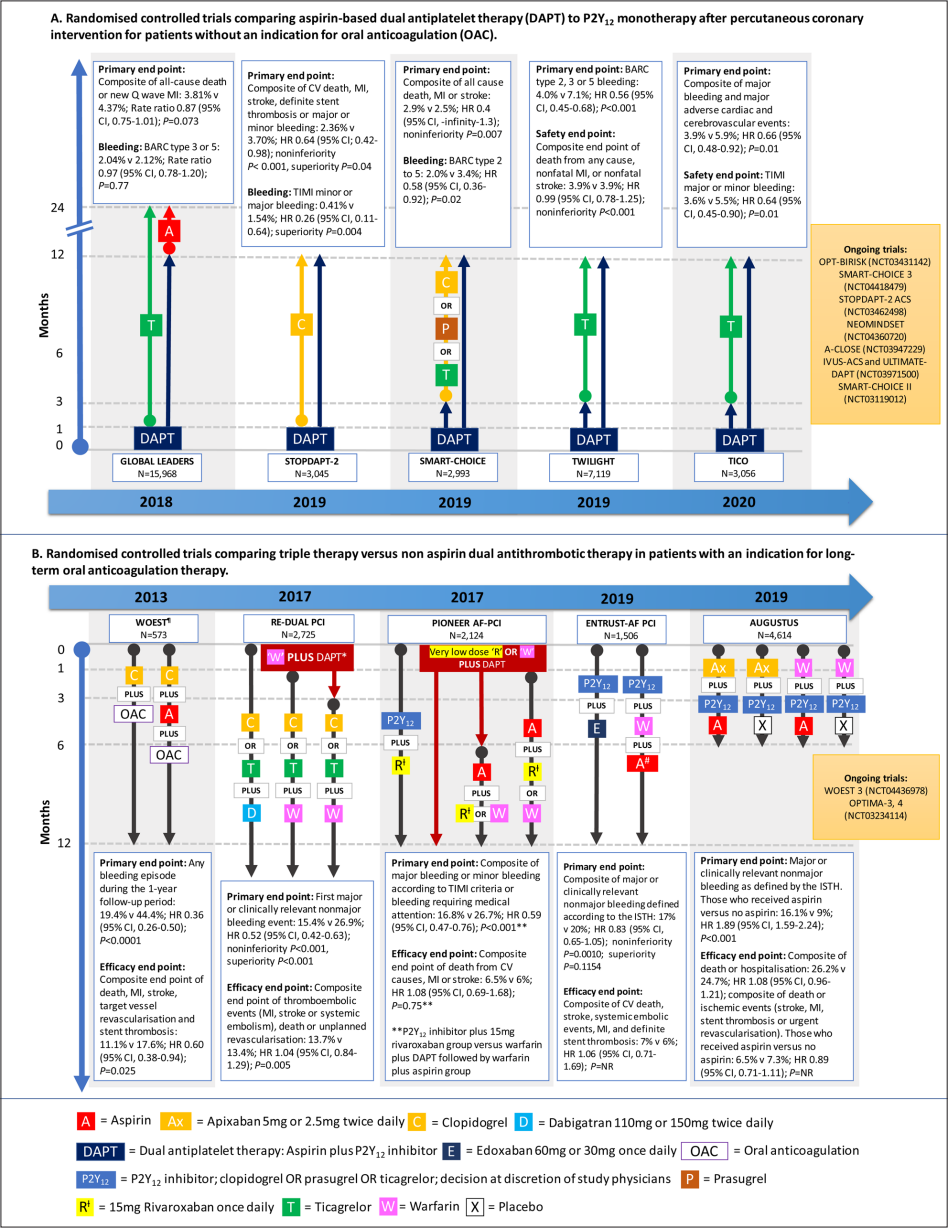
Recent trials assessing aspirin-free strategies following primary percutaneous coronary intervention in patients with and without a dual indication for oral anticoagulation.^5,9^ NR: not reported; HR: hazard ratio; CI: confidence interval; CV: cardiovascular; ISTH: International Society of Thrombosis and Haemostasis; MI: myocardial infarction; BARC: bleeding academic research consortium; TIMI: thrombolysis in myocardial infarction. ¶ Allocated antiplatelet therapy was continued for at least 1 month, up to 1 year in those with stable coronary disease who received a bare metal stent. The decision on duration was at the discretion of the attending physician. In patients with an acute coronary syndrome or those who received a drug-eluting stent, clopidogrel was continued for at least 1 year. * Aspirin was continued for 1 month in those with a bare metal stent and 3 months in those with a drug-eluting stent. # Aspirin was continued for a minimum of 1 month and up to 12 months at the investigator’s discretion. Very–low-dose rivaroxaban = 2.5 mg twice daily.

Two recent meta-analyses including 32,145 patients who underwent PCI in the setting of either CCS or ACS concluded that early aspirin discontinuation (1–3 months after PCI) was associated with a significant reduction in major bleeding by almost 40%, without increasing the ischemic risk or patient mortality.^13,14^ While statistically inconclusive, the meta-analyses do suggest that 3 months of DAPT might be better than 1 month in terms of balancing bleeding and ischemic risks in these post-PCI patients.^13^ A detailed discussion of the individual trials can be found in recent reviews by Jacobsen et al. and Cao et al., with an overview in ***Figure 2***.^5,9^

These findings are now reflected in the most recent guidelines (***Table 1***), which recommend a shortened duration of DAPT, followed by clopidogrel monotherapy without aspirin, as the treatment of choice for those at very high risk of bleeding following PCI.^12,15,16,17,18^ In patients with a concurrent indication for oral anticoagulation (OAC), guidelines also recommend complete discontinuation of all antiplatelets at 12 months while continuing lifelong OAC monotherapy, effectively removing aspirin from the long-term secondary prevention plan in these patients.^15,16^

**Table 1:** Major guideline and consensus recommendations for aspirin use in primary and secondary prevention of cardiovascular disease.*^12,15,16,17,18^ ACS: acute coronary syndrome; AF: atrial fibrillation; ASCVD: atherosclerotic cardiovascular disease; ACC: American College of Cardiology; AHA: American Heart Association; CAD: coronary artery disease; CCS: chronic coronary syndrome; CVD: cardiovascular disease; DAPT: dual antiplatelet therapy; ESC: European Society of Cardiology; MI: myocardial infarction; OAC: oral anticoagulation; PCI: percutaneous coronary intervention; USPSTF: United States Preventive Services Task Force; VTE: venous thromboembolism.


PRIMARY PREVENTION RECOMMENDATION^17^			SECONDARY PREVENTION RECOMMENDATION (FOCUS ON CAD)		
	
GUIDELINE	YEAR	RECOMMENDATION	GUIDELINE	YEAR	RECOMMENDATION

**ESC**	2016*	Not recommended in those with no overt signs of cardiovascular disease (III B).	**ESC**	2019^12^ 2020^16^	***Patients with atrial fibrillation and recent PCI with a concurrent indication for OAC:*** In AF patients with CCS or ACS who undergo uncomplicated PCI, early cessation (≤ 1 week) of aspirin and continuation of dual therapy with an OAC and a P2Y_12_ inhibitor (preferably clopidogrel) for up to 6 or 12 months, respectively, is recommended if the risk of stent thrombosis is low or if concerns about bleeding risk prevail over concerns about risk of stent thrombosis (Class I). OAC monotherapy alone is then continued 12 months post PCI.^16^

**USPSTF**	2016	Recommend aspirin in those aged 50–59 years with ≥ 10% 10-year CVD risk and with no increased bleeding risk (grade: B).	**ACC/AHA**	2016^18^ 2020^15^	In patients treated with DAPT, a daily aspirin dose of 75–100 mg is recommended (1B-NR). Aspirin therapy is almost always continued indefinitely in patients with coronary artery disease.^18^ ***ACC expert consensus decision pathway for anticoagulant and antiplatelet therapy in patients with AF or VTE undergoing PCI or with ASCVD:*** For patients requiring both anticoagulation and antiplatelet therapy, we strongly recommend that the default strategy after recent PCI be dual antithrombotic therapy consisting of anticoagulation and a P2Y_12_ inhibitor (preferably clopidogrel). Anticoagulation monotherapy alone should be continued 12 months post PCI.^15^ Aspirin 75–100 mg for patients with previous MI or revascularization (I A); consider aspirin in CAD patients without a history of MI but with definitive evidence of CAD on imaging (IIb C).^12^

**ACC/AHA**	2019	Consider aspirin use in those aged 40–70 years with higher ASCVD risk and with no increased bleeding risk (IIb A).			


* Guideline update due 2021.

## Primary Prevention

The use of aspirin in primary prevention was motivated by its initial antithrombotic successes in trials of secondary prevention of CVD. However, aspirin’s role in primary prevention has always been controversial.^17^ Aspirin’s first appearance in a major primary prevention guideline was based on five major trials conducted between 1988 and 2001.^19,20,21,22,23^ Further trials followed in the early 2000s.^24,25,26,27,28^ These older trials, summarized in ***Table 2***, were collated in meta-analyses reporting that primary prevention aspirin did reduce nonfatal MI, with a trend to lower mortality, especially in persons with increased CVD risk (eg, 10-yr risk > 10%).^17,20,21,22,23,24,25,26,27,28,29,30,31,32,33,34^ However, the trials conducted in the 2000s suggested that there may be some temporal reduction in aspirin’s efficacy in the primary prevention of CVD, despite constant and unchanging bleeding risks, which prompted further, more contemporary, trials.^17^

**Table 2:** Summary of major primary prevention trials to date. Baseline characteristics represent an average of the complete trial population (ie, both control and treatment arms).^17,20,21,22,23,24,25,26,27,28,29,30,31,32,33,34^ BMD: British Male Doctors; PHS: Physicians Health Study; TPT: Thrombosis Prevention Trial; HOT: Hypertension Optimal Treatment; PPP: Primary Prevention Project; WHS: Women’s Health Study; POPADAD: Progression of Arterial Disease and Diabetes; JPAD: Japanese Primary Prevention of Atherosclerosis With Aspirin for Diabetes; AAA: Aspirin for Asymptomatic Atherosclerosis; JPPP: Japanese Primary Prevention Project; ARRVIE: Aspirin to Reduce Risk of Initial Vascular Events; ASCEND: A Study of CV Events in Diabetes; ASPREE: Aspirin in Reducing Events in the Elderly; TIPS-3: The International Polycap Study-3; BP: blood pressure; CI: confidence interval; CV: cardiovascular; CVD: cardiovascular disease; BP: blood pressure; ABI: ankle brachial index; GI: gastrointestinal; IHD: ischemic heart disease; LDL-c: low density lipoprotein cholesterol; MI: myocardial infarction; NS: nonsignificant; PPI: proton pump inhibitor; SBP: systolic blood pressure; TIA: transient ischemic attack; UA: unstable angina; UK: United Kingdom; US: United States; BMI: body mass index.


STUDY	BMD^17^	PHS^20^	TPT^21^	HOT^22^	PPP^23^	WHS^24^	POPADAD^25^	JPAD^26^	AAA^27^	JPPP^28^	ARRIVE^31^	ASCEND^32^	ASPREE^33^	TIPS-3^34^

Year	1988	1989	1998	1998	2001	2005	2008	2008	2010	2014	2018	2018	2018	2020

No. Participants	5,139	22,071	5,085	18,790	4,495	39,876	1276	2,539	3,350	14,464	12,546	15,480	19,114	5,713

Design	Randomized(computer), unblinded (2:1 randomization in favor of the aspirin group)	Randomized double-blind, placebo-controlled trial 2 × 2 factorial design	Randomized, double-blind, placebo-controlled trial. 2 × 2 factorial design	Prospective randomized double-blind placebo 2 × 2 factorial design	Centrally randomized open-label trial 2 × 2 factorial design	Randomized double-blind placebo- controlled trial 2 × 2factorial design	Randomized double-blind, placebo-controlled trial 2 × 2 factorialdesign	Randomized open-label trial (blinded end point assessment)	Double-blind, randomized controlled trial	Randomized open-label, parallel group	Randomized double-blind, multicenter, placebo-controlled trial	Randomized double-blind, placebo-controlled trial Factorial Design	Randomized double-blind, placebo-controlled trial	Randomized double-blind, placebo-controlled trial with a 2×2×2 factorial design

AspirinDose	300 mg or 500 mg daily aspirin	325 mg every other day	75 mg controlled- release aspirin	75 mg aspirin	100mg enteric- coated aspirin	100 mg every other day	100 mgdaily	81 mg or 100 mg aspirin daily	100 mg aspirindaily	Enteric-coated aspirin 100 mg daily	100 mg enteric-coatedaspirin	100 mg enteric- coated aspirin	100 mg enteric-coatedaspirin	Enteric-coated aspirin 75 mg per day

Comparison	No aspirin	Placebo	Placebo	Placebo	No aspirin	Placebo	Placebo	No aspirin	Placebo	No aspirin	Placebo	Placebo	Placebo	Placebo

Population	Healthy male doctorsin UK between 50–78 years	Healthy male doctors in USages 40–84 years	Men between aged 45 to 69 yearsat high risk for CVD	Men and women aged 50–80years with a diastolic BP between 100 mm Hg and115 mm Hg on two occasions	Men and women ≥ 50with at least one of the major recognized CV riskfactors	Healthy female health professionals ≥45 years	Men and women ≥ 40years with diabetes and ABI ≤ 0.99	Men and women aged30–85 years w/diabetes	Men and women aged 50–75 years w/ABI ≤ 0.95	Men and women aged 60–85 years w/hypertension, hyperlipidemia or diabetes	Men aged ≥ 55 years with 2–4 CV risk factors; women aged ≥ 60 years with ≥ 3 CV risk factors	Men and women aged ≥ 40 years with diabetes	Men and women aged ≥ 70 years	Men aged > 50 years and women aged ≥ 55 years with an elevated INTERHEART score (intermediate or high risk)

**PARTICIPANT CHARACTERISTICS**														

Age (%, mean or median)	< 60 = 47% 60–69 = 39% 70–79% = 14%	40–49 = 41% 50–59 = 34% 60–69 = 19% 70–84 = 7%	Mean: 57.5 years	Mean 62 years	Mean 64 years	Mean55 years	Mean 60 years	Mean 65 years	Mean 62 years	Mean71 years	Mean 64 years	Mean 63 years	65–73 = 49.9% ≥ 74 = 50.1%	Mean 63.9 years

Men	100%	100%	100%	53%	42%	0%	44%	54%	28%	42%	70%	63%	44%	47%

BMI (kg/m2)		≥ 26.4 = 25%	27.4	28.4	27.6	26	29.3	24	-	24.2; BMI > 25 = 79%	28.4; BMI > 25 = 79%	30.7; BMI > 25 = 85%	28.1 BMI > 30 = 30%	25.8

Smoker	31%	11%	41%	16%	15%	13%	31%	21%	33%	13%	29%	8%	3.9	9%

Hypertension	Mean SBP 135.6 mm Hg	Hypertension (39%)	Mean SBP 139 mm Hg	Mean BP 170/105 mm Hg	Mean BP 145/85	Hypertension 26%	Mean BP 145/79 mm Hg	Mean BP 135/77 mm Hg; hypertension 58%	Mean 148/84 mm Hg	Mean BP 137/78 mm Hg; hypertension 85%	Mean SBP 145 mm Hg; hypertension 63%	MeanSBP 136 mm Hg	Hypertension (65%)	Mean SBP 145mmHg

Hyperlipidemia	–	Cholesterol ≥ 6.7mmol/L (4%)	Mean cholesterol 6.4 mmol/L	Mean cholesterol 6.1 mmol/L	Mean cholesterol 6.1 mmol/L	Cholesterol ≥ 6.2 mmol/L or self-reported physician- diagnosed high cholesterol (30%)	Mean cholesterol 5.5 mmol/L	Mean cholesterol 5.2 mmol/L	Mean cholesterol 6.2 mmol/L	Mean cholesterol 5.2 mmol/L	Hyperlipidemia 58% (> 5.2 mmol/L in men; > 6.2 mmol/L in women)	Mean cholesterol 4.2 mmol/L	Mean cholesterol 5.2%; hyperlipidemia 66%	Mean LDL-c 3.1 mmol/L

Diabetes	2%	2%	–	8%	17%	3%	100%	100%	3%	34%	0%	100%	10.8%	36.7%

Statin use (%)	–	–	Potential interaction with warfarin arm, somay have been avoided	–	16%	–	–	26%	Lipid-lowering agents (includingstatins) 4% at start, increased to 25% at 5 years	–	43%	75%	34%	50% (treatment allocation)

PPI (%)	–	–	–	–	–	–	–	–	–	–	–	Approx. 25% at trial completion	25% at trial entry	–

**OUTCOME DATA**														

Follow-up (years)	Median5.5	Median 5	Median 6.8	Mean 3.8	Mean 3.6	Mean 10.1	Median 6.7	Median 4.4	Mean 8.2	Median 5	Median 5	Median 7.4	Median 4.7	Mean 4.6

Primary end point (aspirin vs control)	Definite MI or stroke resulting in death (63.2 vs 62.3per 10,000 person-years; *P*= NS)	CV mortality (81 vs83; RR 0.96; 95% CI, 0.6–1.54)	IHD (154 vs 190events; *P*= .04) Excluding warfarin arm (83 vs 107 events; *P*= NS)	Major CV events excluding silent MI (315 vs368; RR 0.85; 95% CI, 0.73–0.99; *P*= .03)	Major CVevents (45 vs 64; RR 0.71; 95% CI, 0.48–1.04)	MajorCV events (477 vs 522; RR 0.91; 95% CI, 0.80–1.03; *P*= .13)	Major CV (116 vs 117; RR 0.98; 95%CI, 0.76–1.26; *P*= .86) CV death (43 vs 35; RR1.23; 95% CI, 0.79–1.93 *P*= .36)	Major CV events (68vs 86; HR 0.80; 95% CI, 0.58–1.10 *P*= .16)	MajorCV events (13.7 vs 13.3 per 1,000 person-years; HR1.03; 95% CI, 0.84–1.27)	Major CV events (193 vs 207; HR 0.94; 95% CI, 0.77–1.15; *P*= .54)	Major CV events (269 vs 281; HR 0.96; 95% CI, 0.81–1.13; *P*= .60)	Major CV events (658 vs 743; RR 0.88; 95% CI, 0.79–0.97; *P*= .01)	Death, dementia, or persistent physical disability (21.5vs 21.2 per 1,000 person-years; HR 1.01; 95% CI, 0.92–1.11; *P*= .79)	Death from CV causes, MI, or stroke Aspirin vs placebo (116 vs 134; HR 0.86; 95% CI, 0.67–1.10)

Secondary end point (aspirin vs control)	Nonfatal stroke (32.4 vs 28.5 per 10,000 person-years; *P*= NS) and nonfatal MI (42.4 vs 43.3 per 10,000 person-years; *P*= NS)	MI (139 vs 239; RR 0.56; 95% CI, 0.45–0.70; P < .0001) Stroke (119 vs 98; RR 1.22; 95% CI, 0.93–1.60; *P*= .15)	Stroke (47 vs 48; 2.9 vs 3.0 per 1,000 person-years; *P*= NS)	MI (82 vs 127; RR 0.64; 95% CI, 0.49–0.85; *P*= .002) Stroke (146 vs 148; RR 0.98; 95% CI, 0.78–1.24; *P*= .88) CV mortality (133 vs 140; RR 0.95; 95% CI, 0.75–1.20; *P*= .65)	Total CV events (141 vs 187; RR 0.77; 95% CI, 0.62–0.95); CV death (17 vs 31; RR 0.56; 95% CI, 0.31–0.99); All-cause mortality (62 vs 68; RR 0.81; 95%CI, 0.58–1.13)	Fatal MI (14 vs 12; RR 1.16; 95%CI, 0.54–2.51; *P*= .70) Fatal stroke (23 vs 22; RR1.04; 95% CI, 0.58–1.86; *P*= .90) CV death (120 vs126; RR 0.95; 95% CI, 0.74–1.22; *P*= .68)	All-causemortality, nonfatal MI, other vascular events: no significant difference betweengroups	CV mortality (1 vs 10; HR 0.10; 95% CI, 0.01–0.79; *P*= .0037) CHD events (28 vs 35; HR 0.81; 95% CI, 0.49 -1.33; *P*= .40)	Composite of primary endpoint or angina, claudication, or TIA (22.8 vs 22.9 per1,000 person-years; HR 1.00; 95% CI, 0.85–1.17) and all-cause mortality	Composite of primary end point or atherosclerosis (280vs 319; HR 0.89; 95% CI, 0.75–1.04; *P*= .14); CVdeath (58 vs 57; HR 1.03; 95% CI, 0.71–1.48; *P*= .89)	Composite and individual outcomes of the time to CVdeath, MI, or stroke; time to UA, time to TIA, and time to death (*P*= NS for all end points)	Any major vascular event (833 vs 936; RR 0.88; 95%CI, 0.80–0.97); GI cancer (157 vs 158; RR 0.99; *P*= NS)	Major CV events (10.7 vs 11.3 per 1,000 person-years; HR 0.95; 95% CI, 0.83–1.08)	Death from CV causes, MI, or stroke or cancer (153 vs 177; HR 0.86; 95% CI, 0.69–1.07)

Safety end point (aspirin vs control)	Extracranial bleeding (10.6 vs 7.4 per 10,000 person-years; *P*= NS)	Bleeding requiring transfusion (48 vs 28; RR 1.71; 95% CI, 1.09–2.69; *P*= .02)	Major bleeding event (8 vs 4; *P*= NS); intermediate bleeding event (48 vs 33; *P*= NS)	Fatal bleeds (7 vs 8); nonfatal major bleeds (129 vs 70; RR 1.8; P < .001)	Severe bleeding (24 vs 6; P < .0008)	GI bleeding requiring transfusion (127 vs 91; RR 1.40; 95% CI, 1.07–1.83; *P*= .02)	GI bleeding (28 vs 31; RR 0.90; 95% CI, 0.53–1.52; *P*= .69)	Hemorrhagic stroke or severe GI bleeding (10 vs 7; *P*= NS)	Major hemorrhage requiring hospitalization (34 vs 20; HR 1.71; 95% CI, 0.99–2.97)	Extracranial bleed requiring transfusion or hospitalization (62 vs 34; HR 1.85; 95% CI, 1.22–2.81; *P*= .0004)	GI bleeding events (61 vs 29; HR 2.11; 95% CI, 1.36–3.28; *P*= .0007)	Major bleeding event (314 vs 245; RR 1.29; 95% CI, 1.09–1.52; *P*= .003)	Major hemorrhage (8.6 vs 6.2 per 1,000 person-years; HR 1.38; 95% CI, 1.18–1.62; P < .0001)	Major bleeding (21 vs 19), minor bleeding (17 vs 14), and GI bleeding (12 vs 10)


### Recent Trials and Current Guidelines

In 2018, three separate major trials were published that would form the basis of the most recent primary prevention guidelines for aspirin. The Aspirin to Reduce Risk of Initial Vascular Events (ARRIVE) was a pragmatic double-blinded, placebo-controlled, multicenter study that included 12,546 nondiabetic patients with a moderate risk (10–20% 10-year risk) of coronary heart disease.^31^ The study showed no difference in the primary end point of a composite outcome of time to first occurrence of confirmed MI, stroke, CV death, unstable angina, or transient ischemic attack between the two groups (HR 0.96; 95% CI, 0.81–1.13; *P* = .6038). However, on a (less causally valid) per protocol analysis, the hazard ratios for both combined fatal/nonfatal MI and nonfatal MI were lower in the aspirin group (HR 0.53; 95% CI, 0.36–0.79; *P* = .0014 for total MI and HR 0.55; 95% CI, 0.36–0.84; *P* = .0056 for nonfatal MI).

The ASCEND (A Study of Cardiovascular Events in Diabetes) trial included 15,480 participants ≥ 40 years of age, most of whom were considered low (< 5%) to moderate (5–10%) risk for a cardiovascular event in 5 years.^32^ The occurrence of the primary outcome of a first vascular event (a composite of nonfatal MI, nonfatal stroke, or transient ischemic attack, or death from any vascular cause excluding confirmed intracranial hemorrhage) was lower in the aspirin group than the placebo group (8.5% vs 9.6%, respectively; rate ratio 0.88; 95% CI, 0.79–0.97; *P* = .01). There was no significant difference between groups in the mortality rate from all combined vascular causes. The aspirin group had a significantly higher incidence of major bleeding compared with the placebo group (4.1% vs 3.2%; rate ratio 1.29; 95% CI, 1.09–1.52; *P* = .003). Most of these were a result of gastrointestinal bleeding (41.3%).

Aspirin in Reducing Events in the Elderly (ASPREE) was the final and largest of the trials published in 2018.^33^ Relevantly, it targeted an older population with a median age of 74 years. The trial found no significant difference in cardiovascular events (including fatal and nonfatal MI and stroke) between the aspirin versus control groups (HR 0.95; 95% CI, 0.83–1.08), and the rates of fatal CVD were also similar. Significant for this age group, aspirin also did not reduce the risk of incident disability.^35^ In addition, there was a substantially higher rate of major hemorrhagic events in the aspirin group (HR 1.38; 95% CI, 1.18–1.62; *P* < .001) as well as a suggestion of increased mortality (HR 1.14; 95% CI, 1.01–1.29). Following the publication of ASPREE, the American College of Cardiology/American Heart Association recommended aspirin only in select patients and recommend against routine use in primary prevention among adults over age 70.^36^

The most recent study, TIPS-3 (The International Polycap Study 3), was published in 2020.^34^ Participants were randomized in a two-by-two factorial fashion to receive aspirin plus placebo, polypill (simvastatin, atenolol, hydrochlorothiazide, and ramipril) plus placebo, double placebo, or double active treatment. In a direct comparison of aspirin to placebo, the aspirin group showed no difference regarding death from cardiovascular causes, MI, or stroke (HR 0.86; 95% CI, 0.67–1.10). When comparing polypill plus aspirin to double placebo, the primary outcome (a composite of death from cardiovascular causes, MI, stroke, heart failure, resuscitated cardiac arrest, or arterial revascularisation) occurred in 59 (4.1%) of those in the polypill-plus-aspirin group versus 83 (5.8%) in the double-placebo group (HR 0.69; 95% CI, 0.50–0.97). However, given the null finding when comparing aspirin to placebo, this benefit of polypill plus aspirin over double placebo was driven by the polypill component of the intervention. Notably, there were no reported increases in bleeding events in TIPS-3 participants who received aspirin versus placebo. Given the consistency of excess bleeding risks documented across similar previous aspirin trials, this raises questions as to (A) the validity of bleeding outcomes in the TIPS-3 trial, which do not appear to have been adjudicated, and (B) how well participants adhered to the trial regimen. With regard to A, while the aspirin dose was low in TIPS-3 (75 mg), the HOT (Hypertension Optimal Treatment) trial used the same dosing regime in a much larger population and found a significant increase in nonfatal bleeding risks (70 vs 129; RR 1.8; *P* < .001).^23^ With regard to B, there was higher-than-anticipated discontinuation of the trial regime (39.7% for the aspirin vs placebo comparison, whereas expected incidence was 20%). This was partially due to significant trial interruptions from the COVID-19 pandemic, which led to barriers regarding drug delivery, access for in-person follow-up, and overall completion of trial visits. The trial also included a 3- to 4-week run-in period, during which 9.5% of potentially eligible patients were excluded from the randomization process due to intolerance of the trial medications.

### So, What Has Changed?

One of the main theories explaining the potential change in aspirin’s efficacy in CVD prevention focuses on the changing context in which aspirin is being tested. While the most recent aspirin trials attempted to select patients at higher risk, the observed event rates were often lower than expected, likely due to better CVD risk factor management and contemporary treatments.^32,33^ Consequently, the argument has been made that statin use in primary prevention has shown far greater consistent benefit, without the drawbacks of bleeding, and may offer a better “bang for your buck” compared with widespread aspirin use.^37,38^

In addition, the introduction of newer high-sensitivity troponins has changed the way we define MIs. Synchronously, expedited mechanical reperfusion with PCI has now become the standard of care, which means there is a lower likelihood of a fatal outcome after an MI than when original primary prevention trials were conducted. Both of these factors may have changed the influence of aspirin on ischemic outcomes in modern patients.^39^ Dosing regimens of aspirin have also changed significantly, with earlier trials including doses of up to 500 mg compared with 75 mg used in the most recent TIPS-3 trial. Whereas historical trials used “plain” aspirin, newer trials have started to include enteric-coated (EC) aspirin.^5,38^ It is possible that EC aspirin may be less effective than regular aspirin, with suggestions of increased aspirin resistance in those receiving EC aspirin formulations and even reduced oral bioavailability at increased body weights.^38^ All of these factors may be contributing to differences we are seeing in contemporary trial outcomes testing the efficacy of aspirin in primary prevention.

## Where to Next?

### What’s Next in Secondary Prevention?

The recurrent theme from recent secondary prevention trials is that earlier cessation of the aspirin component of DAPT and continuation with a more potent P2Y_12_ inhibitor alone does not translate into an increased ischemic risk in the short term, although it adds the benefit of reduced bleeding risks.^14^ However, most of the trials in this area had a limited follow-up period. Therefore, the question remains as to what should happen once a patient meets the 12-month mark. Is continuation with lifelong P2Y_12_ inhibitors the way forward, or is a switch back to lifelong aspirin and discontinuation of the P2Y_12_ inhibitor the more appropriate choice? Given the increased bleeding risk in the aging population, the question also remains as to whether complete discontinuation of all antiplatelet therapy at 12 months, with focus on maintaining other secondary prevention targets, may also be a worthwhile option.^5^ There are currently several ongoing trials aiming to further elucidate these questions (***Figure 2***).^9^ As it stands, in the absence of a concurrent indication for OAC, aspirin remains the antiplatelet of choice for long-term secondary prevention of ACS/CCS.

### What’s Next in Primary Prevention?

The next frontier for aspirin in the primary prevention space is identifying those individuals considered at high risk of CVD who may benefit from aspirin use as a primary preventive strategy. There are several heterogenous risk scores available, and while they have traditionally performed well in the population in which they were validated, we know that they are imperfect at an individual level.^40,41^

Addition of a coronary artery calcium (CAC) score to personalize a patient’s risk assessment has also been gaining traction, with the 2018 Cholesterol Clinical Practice Guidelines supporting its use in decision making in relation to statin use.^42^ One of the drawbacks of traditional risk factor scores is that patients with higher scores, and therefore considered at higher risk of CVD, are often also at higher risk of bleeding. This is in part based on the heavy reliance on age as a prediction variable, where increasing age confers both an increased risk of CVD and bleeding. While a high CAC also has been correlated with an increased bleeding risk, this correlation is weaker than the association between age and bleeding. Therefore, calculation of a CAC score in those with a borderline or high CV risk score but a lower bleeding risk may help to identify those who will benefit from aspirin therapy. Specifically, CAC scores ≥ 100 and ≥ 400 have been shown to identify those likely to experience a net benefit from aspirin therapy.^43,44^ Conversely, a CAC score equal to 0 may be a useful way of identifying patients with high calculated CVD risk scores who are in fact low risk and in turn should avoid aspirin.^44^

## Conclusion

With rapidly evolving novel antithrombotic and preventive therapies, our ability to modify cardiovascular risk factors has improved. With that, the role of aspirin in both primary and secondary prevention in the modern era also continues to evolve. In secondary prevention, use of P2Y_12_ inhibitors has modified the need for aspirin in patients with higher bleeding risks. Further trials with direct comparisons between the different P2Y_12_ inhibitors and with longer follow-up periods are needed, as are trials truly testing whether PCI can be performed without administering aspirin. In the primary prevention of CVD, newer trials have affirmed that aspirin has a limited role, perhaps best conserved for a select group of primary prevention patients who are at higher risk of CVD but low risk of bleeding. Our task is to identify who exactly these patients are. Furthermore, patients and providers must acknowledge that primary prevention aspirin is only proven to reduce nonfatal CVD events, with no impact on mortality and thus patient longevity. While the landscape is changing, the chapter on aspirin is far from over.

## Key Points

- Rapidly evolving novel antithrombotic and preventive therapies have transformed our ability to modify cardiovascular risk factors. As a result, aspirin’s role in primary and secondary prevention of cardiovascular disease (CVD) is evolving.
- Availability of alternative antiplatelet agents, such as P2Y_12_ inhibitors, mean that aspirin is no longer a ubiquitous requirement beyond the 1-month period following percutaneous coronary intervention (PCI).
- Guidelines now recommend complete discontinuation of aspirin 12 months after PCI in patients with a concurrent indication for oral anticoagulation therapy.
- The use of aspirin for primary prevention may be considered in a select group of patients who are at high risk of incident CVD but have a low bleeding risk.
